# Antidepressant-Like Effect of Selected Egyptian Cultivars of Flaxseed Oil on a Rodent Model of Postpartum Depression

**DOI:** 10.1155/2017/6405789

**Published:** 2017-11-29

**Authors:** Nebal El Tanbouly, Abeer Mohamed El Sayed, Zeinab Y. Ali, Samia Abdel Wahab, Sabah H. El Gayed, Shahira M. Ezzat, Amira Safwat El Senousy, Mouchira A. Choucry, Essam Abdel-Sattar

**Affiliations:** ^1^Department of Pharmacognosy, College of Pharmacy, Cairo University, Cairo 11562, Egypt; ^2^Department of Biochemistry, National Organization for Drug Control and Research (NODCAR), Giza 12553, Egypt; ^3^Department of Pharmacognosy, College of Pharmacy, October University for Modern Sciences and Arts (MSA), 6th of October 12566, Egypt

## Abstract

Flaxseed (*Linum usitatissimum *L.) is a multipurpose crop with health promoting potential. This study was undertaken to investigate the fatty acid profile and yield of fixed oil of six Egyptian flaxseed cultivars. The selected cultivars with the highest content of polyunsaturated fatty acids (PUFAs) (G9 and G10) were assessed for their antidepressant-like effect in rat model of postpartum depression (PPD) induced by hormone-simulated pregnancy followed by hormone withdrawal and compared to fluoxetine. As compared to control group, administration of G9 and G10 (270 mg/kg/day, p.o) for two weeks during the postpartum period can alleviate anxiety and depressive-like behaviors and biochemical changes in PPD-induced rats. This was confirmed by evaluation of anxiety-like behaviors (elevated plus maze, open field test, and forced swim test tests), in addition to biochemical analysis (brain monoamine oxidase-A, corticosterone level, proinflammatory cytokines, and hippocampal redox state). In conclusion, flaxseed oil of Egyptian cultivars G9 and G10 exhibited significant antidepressant-like effect in rat model of PPD without affecting locomotor activity. At the treatment doses, the antidepressant-like activity of Giza 9 oil is comparable to fluoxetine.

## 1. Introduction

According to World Health Organization [1], the postnatal period is a very difficult transition phase for a mother, where she has to face a lot of changes, physically and emotionally. It is a severe condition which may has serious consequences for the whole family and not only the mother or her child. WHO recommended guidelines focused on the six weeks after delivery care including hemorrhage, infections, anemia, and depression [2]. WHO are updating their guidelines on regular basis, and the next update will be in 2018. Postpartum depression (PPD) is one of the major depressive disorders that occurs within the first month after childbirth [2].

Postpartum depression (PPD) occurs in 10–15% of childbearing women [3]. It causes significant morbidity in postpartum women and their newborns. PPD is accompanied by headache, exhaustion, mood swings, irritability, anxiety, and anhedonia [4], and in severe cases the mother can harm herself or her baby [5, 6].

Weigelt et al. (2013) reported a hypothesis based on both experimental and clinical evidences that the immune dysregulation is involved in the development of postpartum depression [7]. It was observed that the level of anti-inflammatory cytokines responsible for suppression of immune function was elevated, where the proinflammatory cytokines such as tumor necrosis factor- (TNF-) *α* and interleukin- (IL-) 6 are downregulated. Upon delivery the state of the immune system was reversed into a higher level of proinflammatory cytokines, which lasts for several weeks. It is commonly known that higher levels of tumor necrosis factor- (TNF-) *α* and interleukin- (IL-) 6 were reported in patients with depression [8]. Inconsistent results and controversial studies failed to confirm the relationship between immune dysregulation and PPD and a better understanding of the pathophysiology of PPD is needed [9, 10]; thus, the role of immune function in PPD needs further studies [11].

Nutritional status also plays a role in mental health and nutritional intervention may improve the postpartum depression. The supplementation with omega-3 polyunsaturated fatty acids (*ω*-3 PUFAs) rich products in such conditions is of great value, because *ω*-3 PUFAs aids in membrane integrity and fluidity, which is pivotal for neurotransmitter binding and signaling within the cell, which are dysfunctional in depressive patients [12]. The proinflammatory markers such as IL-1*β*, IL-2, IL-6, interferon-*γ*, and TNF-*α* are expressed in the PPD model of rats. A number of studies have shown a relationship between these cytokines and the development of depression through alteration of the metabolism of neurotransmitters and neurotransmitter transporter mRNAj or their precursors availability [13, 14].

*Linum usitatissimum* L. (Linaceae), commonly known as flaxseed or linseed, is known to be the richest plant source of *α*-linolenic acid (ALA), attaining up to 55% of the total fatty acids [15]. A portion of ALA converted in the body to eicosapentaenoic acid (EPA), docosahexaenoic acid (DHA), though the actual converted percentage may differ between men and women [16]. The human body requires a variety of *ω*-3 PUFAs. Since ALA is a precursor to EPA and DHA, so flaxseed oil can be considered as an alternative cheap omega-3 source compared to fish oil, especially for vegetarians.

Among the biological activities of omega-3 PUFAs is their well-documented anti-inflammatory properties, which should be considered to understand the mechanism by which they treat or prevent depression [17, 18]. Despite the belief of importance of omega-3 PUFAs for the nervous system and neuronal dysfunction, the underlying mechanism remains unclear. Numerous studies have shown that deficiency in omega-3 leads to neuronal dysfunction and change in the inflammatory status but results are still inconclusive regarding the association between omega-3 PUFAs and PPD. Su et al. (2015) reported the role of omega-3 polyunsaturated fatty acids in prevention of mood and anxiety disorders such as major depression, bipolar disorders, stress disorder, and other depression related diseases [19].

Therefore, the current study aimed (i) to study the comparative composition of the fatty acid profiles of six selected Egyptian flaxseed cultivars and (ii) to test the hypothesis that the highest cultivars in omega-3 fatty acids G9 and G10 could attenuate the biochemical changes and depression symptoms in rat model of PPD compared to the antidepressant drug fluoxetine (FLX).

## 2. Materials and Methods

### 2.1. Plant Material

Samples of* L. usitatissimum *seeds were collected in winter of 2012-2013 from different localities in Egypt, namely, Qalyubia, Sharqia, Gharbia, and Kafr El-Sheikh Governorates, and identified by staff members of Fiber Crops Research Department, Faculty of Agriculture, Giza. Six flax cultivars, namely, Sakha 1 (S1), Sakha 2 (S2), Sakha 3 (S3), Sakha 4 (S4), Giza 9 (G9), and Giza 10 (G10), were used in this study. Specimens of the seeds were deposited in the Herbarium of Pharmacognosy Department, Faculty of Pharmacy, Cairo University (9-11-2015, 1-6).

### 2.2. Chemical Assessment

#### 2.2.1. Investigation of the Lipoidal Content

One hundred grams of the powdered seeds of each flaxseed cultivar (S1, S2, S3, S4, G9, and G10) was exhaustively extracted by* n*-hexane with the aid of sonication three times each for 30 min with 500 ml* n*-hexane. The extract was evaporated under reduced pressure at a temperature not exceeding 50°C, to yield an oily liquid. An oil sample (2 g) of each cultivar was saponified with 20% ethanolic KOH for four hours and the saponifiable and unsaponifiable fractions were separated [20]. The saponifiable fraction as well as the available authentic samples of fatty acids were methylated with diazomethane [20] and subjected to GC analysis.

#### 2.2.2. Investigation of the Saponifiable Matter (SM)

GC-MS of fatty acids: GC/MS analysis was carried out on Hewlett Packard HP 6890 Series Gas Chromatograph System with a HP 5973 Mass Selective Detector, operating with the following parameters: Column TR-FAME (Thermo 260 M142 P) (30 m, 0.25 mm ID, and 0.25 m*υ* Film) (70% cyanopropyl polysilphphenylene siloxane) capillary column. The carrier gas was helium, flow rate (1.5 ml/min). Split injection was conducted with a split ratio of 10 : 1. Injector temperature was 200°C. Detector temperature was 280°C, with temperature programming as follows: initial temperature 80°C, hold for 2 min, ramp to 230°C at 3°C/min, and hold time for 5 min. The amount of sample injected was about l *μ* l (conc 5 *μ*l sample per 1 ml solvent). The mass spectrometer was operated in electron-impact (EI) mode. Precolumn pressure was 70 kPa. Injection temperature was 250°C. Ion source was EI (200°C). Interface temperature was 280°C. Electron energy was 70 eV. Solvent delay was 5.5 min. For qualitative analysis, the full scan mode was used and the scan range was 40–400 *m*/*z*. Qualitative identification of the different constituents was performed by comparison of their relative retention times and mass spectra with those of authentic reference compounds (fatty acid methyl esters, purity 98% by GC). Also, probability merge search software and the NIST MS spectra search program were used. The results of oil yield and fatty acid profile of the investigated oil sample are presented in Table 1.

### 2.3. *In Vivo* Assessment of Antidepressant Potential of Selected Cultivars of Flaxseed Oil in a Rat Model of Postpartum Depression

#### 2.3.1. Animals

A total of 40 adult Sprague-Dawley female rats (3 months old) weighing 160 ± 10 g were provided from the animal house of National Organization for Drug Control and Research (NODCAR), Giza, Egypt. They were kept under standard conditions with temperature at 23 ± 2°C and 12/12 hours light/dark cycle and allowed free access to normal food and water throughout the experiment. The experimental design was conducted in accordance with the guidelines of the National Institutes of Health Guide for Care and Use of Laboratory Animals (Publication number 85-23, revised 1985). The protocol was approved by the Institutional Animal Care and Use Committee (IACUC, number 9-031), Faculty of Pharmacy, Cairo University.

#### 2.3.2. Induction of PPD in Rats

*(1) Surgery.* At the beginning of the experiment, rats were subjected to bilaterally ovariectomized (OVX) by using an aseptic technique under anesthesia with thiopental (50 mg/kg i.p.). The OVX rats were allowed to recover for one week following surgery [21].

*(2) Hormone-Simulated Pregnancy Regimen*. Hormonal withdrawal model represents a valid approach for the study of certain aspects of PPD, as it captures the possible negative impacts of hormonal fluctuations associated with pregnancy and lactation on emotion regulation [22]. In the present study, one week after recovery from surgery, OVX rats were subjected to hormone-simulated pregnancy (HSP) regimen. OVX rats were s.c injected with a low dose of estradiol benzoate (Sigma, St. Louis, MO) (2.5 *μ*g/rat) and a high dose of progesterone (Sigma, St. Louis, MO) (4.0 mg/rat) daily for 16 consecutive days. On days 17–22, a higher dose of estradiol benzoate (50 *μ*g/rat) only was given to mimic the levels observed in the actual rat's pregnancy. Hormone and vehicle treatments ceased after day 22, initiating the hormone withdrawal period. The days after hormone termination, mimicking the postpartum drop in gonadal hormones is considered as the postpartum period [3, 21].

#### 2.3.3. Experimental Design

**Figure 1:**
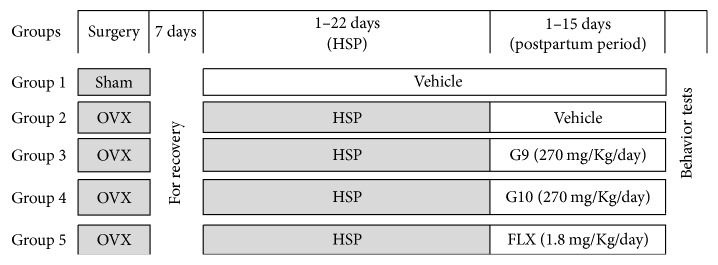
Diagram showing the experimental design and different treatments. OVX: ovariectomized; HSP: hormone-simulated pregnancy; G9 and G10: flaxseed oils from cultivars Giza 9 and Giza 10; and FLX: fluoxetine.

A total number of 40 adult female rats were divided randomly into 5 equal groups each containing eight rats (Figure 1).* Group 1:* sham-operated animals received vehicle (safflower oil) on the same schedule and served as control.* Group 2:* PPD- induced rats were untreated and served as positive control for two weeks.* Groups 3-4:* PPD-induced rats received flaxseed oil (G9 and G10) at a fixed dose of 270 mg/Kg b.wt/day (equivalent to human dose of 3000 mg/day) for two weeks.* Group 5:* PPD-induced rats received a reference antidepressant drug FLX at a dose of 1.8 mg/Kg b.wt/day (equivalent to human recommended dose of 20 mg/day) for the same period [23].

At the end of the treatment period, all animals were subjected to behavior tests. Blood samples were collected from retro-orbital plexus after overnight fasting. Sera were separated by centrifugation at 640*g* and 4°C for 15 minutes and stored at −20°C for different biochemical analysis. All rats were sacrificed and the brains were dissected out rapidly and rinsed in ice-cold saline. Hippocampi were quickly dissected on an ice surface homogenized in saline, centrifuged at 640*g* and 4°C for 15 minutes, and the obtained supernatants were stored at −80°C. Furthermore, spleen and thymus were harvested and weighed in relation to body weight to determine immune organ index.

#### 2.3.4. Behavioral Tests

Anxiety-like behaviors were evaluated by the elevated plus maze (EPM) test and open field test (OFT), whereas depression-like behaviors were assessed by the forced swim test (FST).

*(1) Elevated Plus Maze Test (EPM)*. Elevated plus maze (EPM) is commonly used to assess anxiety-like behavior and to evaluate learning and memory in laboratory animals [24]. The apparatus consists of two open arms (50 × 10 cm) and two closed arms (50 × 10 × 40 cm) extended from a central platform (10 × 10 cm). The maze is elevated to a height of 40 cm from the floor. Two separated experiments were conducted in EPM. The first one was performed in two stages (learning and acquisition). On the first day (training session), each rat was placed at the end of an open arm facing away from the center. The time in seconds taken to enter any one of the closed arms with its four legs was recorded as transfer latency (TL). Retention testing was conducted 24 h after the first trial and recorded in a similar manner as mentioned before. Significant decrease in TL value of retention was considered as an index of improvement in memory. In the second experiment, each animal was placed in the center of the maze facing a closed arm and the cumulative time spent in the open/closed arms was recorded through a 5 min session. An arm entry was defined when four paws of the rats were inside the arm [25].

*(2) Open Field Test*. Locomotor activity was assessed by open field test (OFT) according to the method of Stout and Weiss (1994) [26]. Rats were individually housed in a rectangular container made of dark polyethylene (60 × 60 × 30 cm) to provide best contrast to the white rats in a dimly lit room equipped with a video camera above the center of the room, and their locomotion and exploratory behaviors were then measured. The open field maze was divided into two zones, central and peripheral zone, using the square drawn on the maze. The area was divided into 16 squares of 15 × 15 cm by painted white lines. Each rat placed in one corner of the open filed and its activity during the subsequent 5 min was recorded. The behavioral parameters assessed were (1) ambulation (number of squares traversed by rat); (2) rearing frequency (number of times the animal stood on its hind legs); and (3) grooming (duration of time the animal spent licking or scratching itself while stationary).

*(3) Forced Swim Test (FST).* Forced swim test (FST) was performed according to a standard method of Lucki (1997) to determine the antidepressant-like behavior [27]. The test apparatus consisted of a vertical cylindrical glass container (46 cm high, 21 cm in diameter) filled to a depth of 30 cm with tap water at 25 ± 0.5°C. This depth was sufficient to ensure that animals could not touch the bottom of the container with their hind paws or their tails. The rats had a swimming-stress session for 15 min and then were removed. The water was changed for each rat. The next day, all animals were subjected to 5 min of forced swimming and the three behaviors recorded were (1) climbing behavior, which is defined as upward-directed movements of the forepaws along the side of the swim chamber; (2) swimming behavior, the movement (usually horizontal) throughout the swim chamber that also includes crossing into another quadrant; and (3) immobility, which was assigned when no additional activity was observed other than that required to keep the rat's head above the water.

#### 2.3.5. The Immune Organ Index

The initial and final body weight and thymus and spleen weights were recorded. Thymus and spleen indexes were calculated according to the following formula: thymus or spleen index (mg/g) = weight of thymus or spleen (mg)/body weight of rat (g) [28].

#### 2.3.6. Biochemical Analysis

The clinical diagnostics were determined in accordance with the manufacturers' instructions of the corresponding kit. 

*(1) Assay of Monoamine Oxidase and Corticosterone*. Brain monoamine oxidase-A (MAO-A) was investigated according to the spectrophotometric method of Charles et al. (1977) [29], while serum corticosterone (CORT) level was determined quantitatively using rat ELISA kits (DRG International, USA) [30]. 

*(2) Assay of Proinflammatory Cytokines.* The serum levels of proinflammatory cytokines were determined by using rat ELISA kits for TNF-*α* (IBL International, Hamburg, Germany) and IL-1*β* and IL-6 (R&D Systems, Minneapolis, MN, USA). 

*(3) Quantitative Determination of Antioxidant Defense System Markers. *Superoxide dismutase (SOD; EC 1.15.1.1) and catalase (CAT; EC 1.11.1.6) were determined by the method of S. Marklund and G. Marklund (1974) [31] and Aebi (1984) [32], respectively. Reduced glutathione (GSH) and vitamin C content were determined by the method of Ellman (1959) [33] and Omaye et al. (1979) [34], respectively. Protein content of hippocampal supernatant was determined calorimetrically using method developed by Lowry et al. (1951) [35] using bovine serum albumin as standard. 

*(4) Quantitative Determination of Oxidative Stress Markers.* Malondialdehyde (MDA), a marker of lipid peroxidation, was assayed using thiobarbituric acid reacting substance (TBARS) [36]. As a hallmark of protein oxidation, total protein carbonyl (PC) content was determined by the method described by [37]. The level of the nitric oxide (NO) was estimated as nitrate/nitrite by Griess reaction after conversion of nitrate to nitrite according to the method of Montgomery and Dymock (1961) [38].

### 2.4. Statistical Analysis

The obtained biochemical data are expressed as means ± SE and subjected to statistical analysis by one-way analysis of variance (ANOVA) using the Statistical Package for the Social Sciences (SPSS Inc., Chicago, USA, version 22 software) followed by Duncan's multiple-comparison test. Values of *P* < 0.05 are considered to indicate statistical significance.

## 3. Results

### 3.1. Chemical Assessment (Fatty Acids Profile)

**Figure 2:**
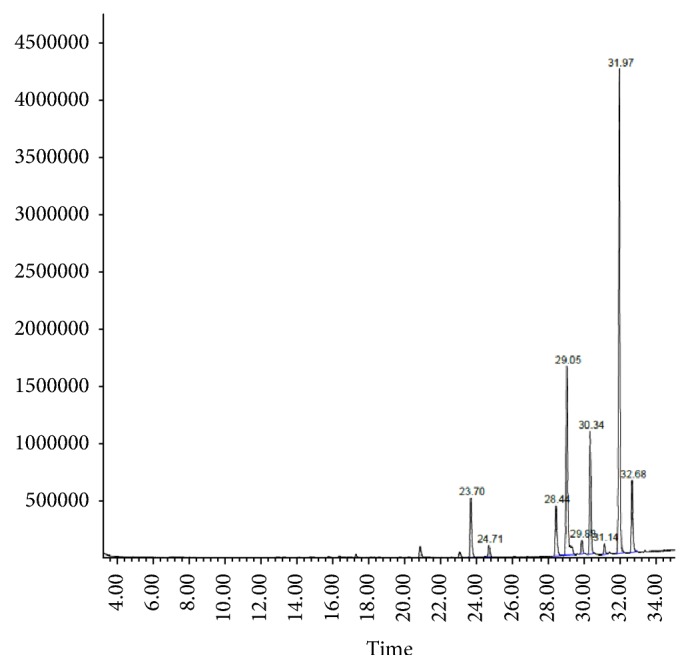
GC chromatogram of fatty acid profile of flaxseed oil cultivar G9.

**Figure 3:**
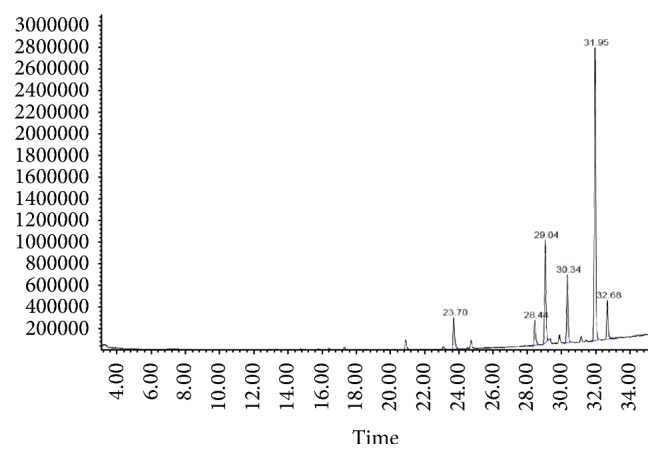
GC chromatogram of fatty acid profile of flaxseed oil cultivar G10.

The fixed oil yield of six different Egyptian cultivars of flaxseed (Table 1) ranged from 33.2 to 42.5%. Fatty acid profiles of investigated samples were similar qualitatively and vary quantitatively among the examined flaxseed oil cultivars. Comparative GC/MS analysis of the fatty acid profile of the tested oil cultivars (Figures 2 and 3 and Table 1) revealed the detection of ten fatty acids, among which the unsaturated fatty acid constituted about 87.73–90.71% of the total composition. GC/MS analysis prevailed that all the tested samples of flaxseed oil cultivars contain comparable amounts of the *ω*-3, *ω*-6, and *ω*-9 PUFAs. The unsaturated fatty acids were dominated by *α*-linolenic acid (53.31–59.04%) which represented the *ω*-3 fatty acid followed by oleic acid (14.13–22.12%) which represented *ω*-9 fatty acid, while *ω*-6 unsaturated fatty acids were represented by linoleic acid (0.74–10.52%). The major identified saturated fatty acids were palmitic and stearic acids. Fixed oil derived from Giza 9 (G9) and Giza 10 (G10) contains the highest percentage of *ω*-3 (59.04 and 53.88%, resp.), while the lowest percentage was detected in Sakha 1 (S1).

### 3.2. Evaluation of the Antidepressant Potential of Selected Cultivars of Flaxseed Oil in a Rat Model of Postpartum Depression

#### 3.2.1. Elevated Plus Maze Model (EPM)

The results in Table 2 demonstrate a behavioral phenotype that is relevant to PPD symptoms, including increased anxiety and learning and memory diminishing properties. The obtained results showed that the prolongation of transfer latency (TL) in the PPD rats was reversed by treatment with flaxseed oil (G9 and G10) and FLX for 2 weeks during postpartum period.

**Figure 4:**
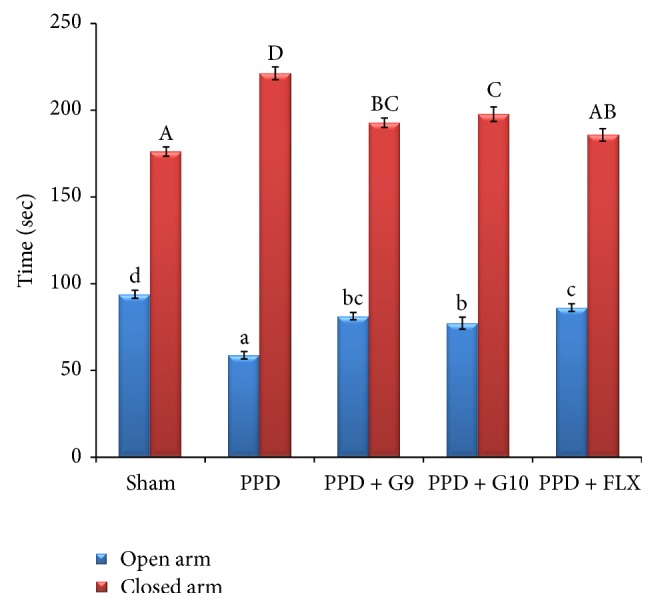
Time spent in open and closed arms on the elevated plus maze test. Each value represents the mean of 8 rats ± SE. The different superscripts (a–d for open arm and A–D for closed arm) indicate a significant difference between groups using one-way ANOVA using SPSS software (version 22) followed by Duncan's multiple-comparison test at *P* < 0.05. PPD: postpartum depression; G9 and G10: flaxseed oil from cultivars Giza 9 and Giza 10; and FLX: fluoxetine. PPD exhibited significant decrease in the time spent in open arm and increase in the time spent in closed arm versus sham-operated group. However, treatment with either flaxseed oil cultivars (G9 and G10) or fluoxetine significantly increased the time spent in open arm in order of FLX ≥ G9 > G10 and decreased the time spent in closed arm in order of FLX > G9 ≥ G10 versus PPD group.

During the elevated plus maze (Figure 4), PPD rats spent significantly (*P* < 0.05) more time in the closed arms (221.3 ± 3.68 second) in comparison to control rats (176.1 ± 2.70 second). Both of flaxseed oil (G9 and G10) and FLX significantly (*P* < 0.05) decreased the time spent in the closed arms, reflecting a reduction in anxious behavior. Conversely, the time spent of PPD rats in the open arms (58.8 ± 2.14 seconds) were significantly (*P* < 0.05) increased by flaxseed oil (G9 and G10) and FLX treatment as compared to control animals.

#### 3.2.2. Open Field Test (OFT)

**Figure 5:**
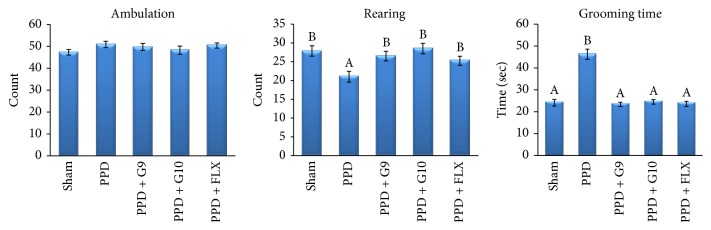
Effect of different treatments on ambulation, rearing, and self-grooming behavior in open field test. Each value represents the mean of 8 rats ± SE. The different capital letters indicate a significant difference between groups using one-way ANOVA by using SPSS software (version 22) followed by DMCT Duncan's multiple-comparison test at *P* < 0.05. PPD: postpartum depression; G9 and G10: flaxseed oils from cultivars Giza 9 and Giza 10; and FLX: fluoxetine.

During the open field test (Figure 5), untreated PPD significantly (*P* > 0.05) decreased the numbers of rearing and increased the time of self-grooming by 24.7% and 91.7%, respectively, versus control group. It is important to notice that no significant differences (*P* > 0.05) were observed in ambulation between all animal groups. However, a significant (*P* < 0.05) increase in numbers of rearing and decrease in grooming time were observed in PPD-treated groups with either FLX or flaxseed oil (G9 and G10) versus sham group.

#### 3.2.3. Forced Swim Test (FST)

**Figure 6:**
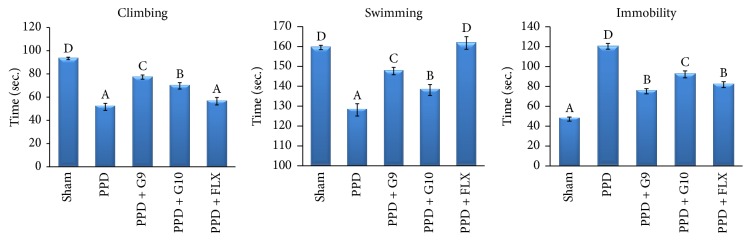
Effect of different treatments on climbing, swimming, and immobility in forced swim test. Each value represents the mean of 8 rats ± SE. The different capital letters indicate a significant difference between groups using one-way ANOVA by using SPSS software (version 22) followed by DMCT Duncan's multiple-comparison test at *P* < 0.05. PPD: postpartum depression; G9 and G10: flaxseed oils from cultivars Giza 9 and Giza 10; and FLX: fluoxetine.

The data in Figure 6 revealed that animals subjected to induction of PPD exhibited a depression-like behavior in FST, characterized by significant (*P* < 0.05) increase in duration of immobility by 155.2% and decrease in active swimming and climbing by 19.7% and 44.7%, respectively, versus sham-operated group. In contrast, daily treatment with the flaxseed oils of G9 or G10 at a fixed dose of 270 mg/Kg b.wt for 15 days displayed a significant (*P* < 0.05) decrease in durations of immobility by 37.6% and 23.4%, respectively, versus untreated PPD rats. Treatment with FLX significantly (*P* < 0.05) decreased the immobility by 32.0% and increased the active swimming time by 26.3% but insignificantly (*P* > 0.05) affected climbing duration versus untreated PPD rats. In contrast, flaxseed oil (G9 and G10) supplementation significantly (*P* < 0.05) increased the climbing time by 49.7% and 35.2%, respectively, during 5 min, versus untreated PPD rats.

### 3.3. Effect of Different Treatments on Body Weight and Immune Organ Index

The results depicted in Table 3 revealed that the weight gain significantly decreased (*P* < 0.05) by 2.2-fold in untreated PPD-induced female rats as compared with sham-operated rats. Treatment of PPD rats with flaxseed oils (G9 and G10) and FLX exhibited a significant increase (*P* < 0.05) in body weight gain by 2.0-, 1.97-, and 2.2-fold, respectively, versus untreated PPD rats. In addition, Table 3 elucidated that, in PPD rats, thymus and spleen indexes were significantly (*P* < 0.05) decreased by 14% and 17%, respectively, as compared with sham-operated rats, while they were significantly increased in PPD rats receiving either G9, G10 flax oils, or FLX by 11, 10, and 13%, respectively, for thymus, and by 15, 9, and 17%, respectively, for spleen versus untreated PPD-induced female rats.

### 3.4. Effect of Different Treatments on Brain Monoamine Oxidase, Serum Corticosterone, and Proinflammatory Cytokines

Data in Table 4 revealed that, in PPD rats, the levels of brain MAO-A enzyme activity, serum levels of CORT, and proinflammatory cytokines TNF-*α*, IL-1*β*, and IL-6 were significantly (*P* < 0.05) increased by 1.47-, 1.92-, 2.0-, 1.95-, and 3.93-fold, respectively, versus sham-operated rats. Conversely, MAO-A activity and CORT were significantly decreased in rats receiving G9, G10 flaxseed oils or FLX by 25.5, 13.9, and 20%, respectively, for MAO-A, and by 31, 29, and 33%, respectively, for CORT versus untreated PPD rats. Also, treatment of PPD rats with G9, G10, or FLX levels for 15 days significantly suppressed serum levels of proinflammatory cytokines by 42, 39, and 43% and TNF-*α*, by 34, 34, and 38% for IL-1*β*, respectively, and by 52, 48, and 59%, respectively, for IL-6 versus untreated PPD rats.

### 3.5. Effect of Different Treatments on the Hippocampal Redox State

**Figure 7:**
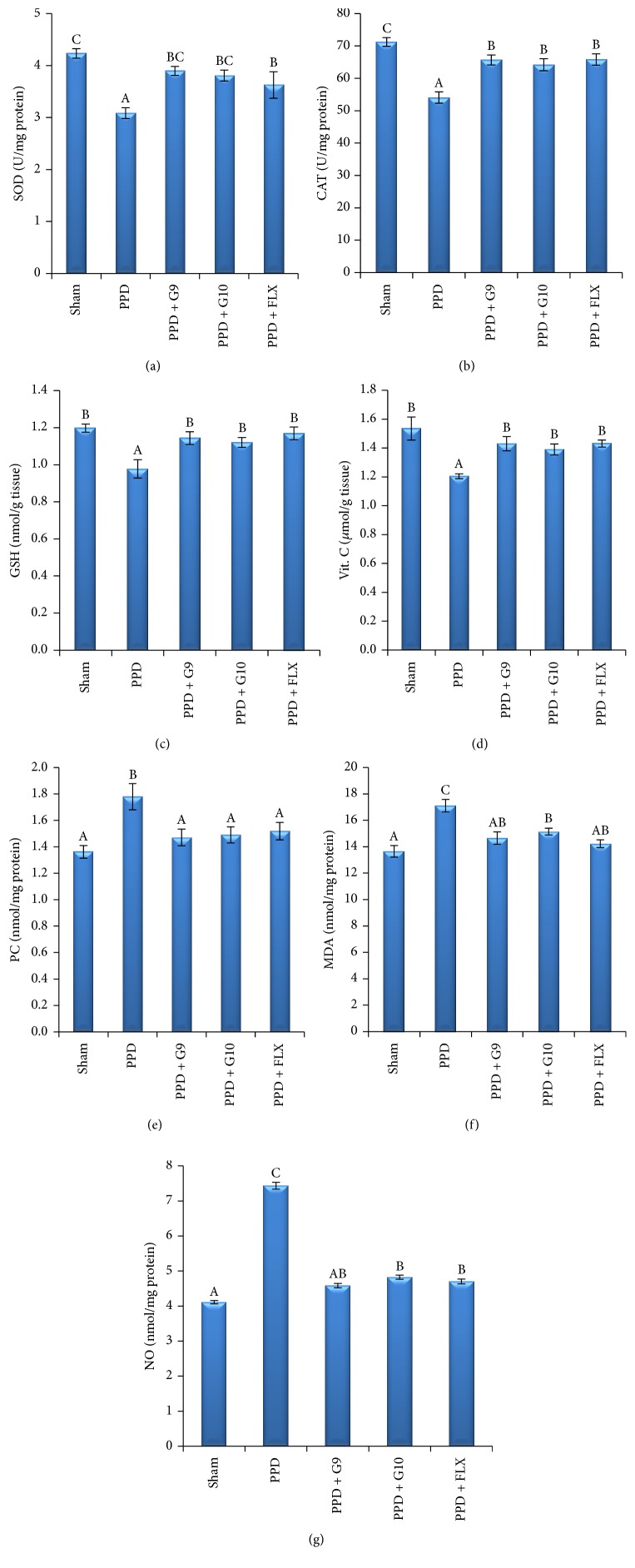
Effect of different treatments on the hippocampal redox state. Each value represents the mean of 8 rats ± SE. In the same column, the different letters indicate a significant difference between groups using one-way ANOVA by using SPSS software (version 22) followed by Duncan at *P* < 0.05. Superoxide dismutase (SOD, U/mg protein); catalase (CAT, U/mg protein); reduced glutathione (GSH, nmol/mg protein); vitamin C (vit C, *μ*mol/g tissue); protein carbonyl (PC, nmol/mg protein); malondialdehyde (MDA, nmol/g tissue); and nitric oxide (NO, *μ*mol/mg protein). One unit of SOD corresponds to the enzyme required to inhibit half of the oxidation of pyrogallol; one unit of CAT is defined as the amount of enzymes required to decompose 1 m mole of hydrogen peroxide in 1 min.

The obtained results illustrated in Figures 7(a)–7(e) and Table 5 illustrated that the hippocampal SOD, CAT, reduced GSH, and vitamin C contents were significantly decreased by 27, 24, 18, and 21%, respectively, while hippocampal PC, MDA, and NO contents (Figures 7(e)–7(g)) were significantly increased by 31, 25, and 81%, respectively, in PPD rats versus sham group. On the contrary, hippocampal SOD and CAT enzyme activity were significantly increased in rats administrating G9, G10, or FLX by 26, 23, and 17%, respectively, for SOD, and by 21, 19, and 22%, respectively, for CAT against untreated PPD rats. Also, treatment of PPD rats with G9, G10, and FLX levels significantly increased the hippocampal reduced GSH and vitamin C content by 16, 14, and 19%, respectively, for GSH and by 18, 15, and 18%, respectively, for vitamin C versus untreated PPD rats. Furthermore, hippocampal PC, MDA, and NO content was significantly decreased in rats treated with G9, G10, or FLX treated PPD rats to 17, 16, and 15%, respectively, for PC, by 14, 11, and 17%, respectively, for MDA, and by 38, 35, and 37%, respectively, for NO versus untreated PPD group.

## 4. Discussion

### 4.1. Chemical Assessment (Fatty Acids Profile)

Flax cultivars have been evaluated regarding yield, yield components, quality of fibers, and seeds in addition to oil chemical composition [39]. Many investigators indicated significant differences among flax genotypes [40]. Flaxseed is the richest source of *α*-linolenic acid (ALA), with five times more ALA than any other plant food. The relative high percentage of unsaturation in fatty acids especially omega-3 is beneficial as a food supplement in treating hyperlipidemia and/or hypercholesterolemia [41].

The fixed oil yield of the examined Egyptian cultivars (Table 1) ranged from 33.2 to 42.5%, which are nearly similar to the reported data (38 to 44%) due to genotype and environmental parameters variation [42]. Fatty acid profiles of the oil samples were similar qualitatively and vary quantitatively among the examined flaxseed oil cultivars. GC/MS analysis showed that all the tested samples of flax oil cultivars contain comparable amounts of the *ω*-3, *ω*-6, and *ω*-9 of unsaturated fatty acids. Results of the fixed oil derived from the Egyptian cultivars are in agreement with the previously reported data of Bartram (2013) [15]. The highest percentage of *ω*-3 was detected in the oil samples derived from Giza 9 and Giza 10 cultivars (59.04 and 53.88%, resp.). Oleic acid (*ω*-9) was detected in the highest percentage in oil derived from Giza 10. Oil samples derived from Giza 9 (G9) and Giza 10 (G10) cultivars were found to contain a relative percentage of unsaturated fatty acid *ω*-3 (53.30–58.48%), *ω*-6 (12.30–11.87%), and *ω*-9 (20.84–18.54%), respectively. Moreover, the higher ratio of omega-3 fatty acids (ALA) relative to omega-6 (linoleic acid) is very important for prostaglandins metabolism which in turn pivotal in the regulation of inflammation, hormone synthesis, and steroid production [43].

A previous study demonstrated the antidepressant effects of omega-3 fatty acid in postpartum model of depression in rats [3]; however, no studies are available to suggest the benefits of flaxseed oil for improving mood even in the general population. Therefore, we test the hypothesis that daily oral administration of oils of Egyptian cultivars of flaxseed G9 and G10 containing the highest percentage of *ω*-3 would exhibit antidepressant-like effect by amelioration of the behavior and biochemical changes in PPD model female rat.

### 4.2. Biological Assessment of the Antidepressant-Like Effect of Flaxseed Oil of Selected Cultivars Using Postpartum Depression (PPD) Model in Rat

Anxiety and depression-like behaviors are associated with postpartum depressive-like symptoms [44]. In the present study, the behavioral responses of PPD rats treated with flaxseed (G9 and G10), compared with FLX as a standard antidepressant drug, were assessed in EPM test, OFT, and FST, commonly considered as standard models of depression in animals.

#### 4.2.1. Effect of Flaxseed Oil Cultivars on PPD Rat Behavior

EPM served as the behavioral model to evaluate anxiety and learning and memory improvement properties [3, 28]. In the present study, HSP induced depressive-like behavior in PPD rats is manifested by increased transfer latency (TL) value of learning and retention (Table 2) and increase in the time spent in closed arm (Figure 4) in EPM test. Administration of flaxseed oil or FLX significantly decreased TL values of learning and retention, indicating better memory retention and significant memory improvement as compared with untreated PPD group.

EPM task in the second experiment is assessing the anxiety-like behaviors in rodents. The obtained data in Figure 4 revealed that PPD rats tend to spent more time in closed arm. The task is based on an approach-avoidance conflict, meaning that the animal is faced with a struggle between a propensity to discover a new environment and an unconditioned fear of high and open places. Treatment with either flaxseed oil (G9 and G10) or FLX significantly decreased the time spent in the closed arms, while it increased the time in open arm reflecting a reduction in anxious behavior or antianxiety behavior. These beneficial effects were decreased in the order of FLX ≥ G9 > G10 in treated groups.

OFT provides simultaneous measures of locomotion, exploration, anxiety, and emotionality by observing the ambulation, rearing, and self-grooming behaviors. A high frequency of these behaviors indicates increased locomotion and exploration and/or a lower level of anxiety [45]. In the present study, untreated PPD significantly decreased the numbers of rearing and increased the time of self-grooming versus control group (Figure 5). However, a significant increase in numbers of rearing and decrease in grooming time without affecting locomotion activity were observed in PPD-treated groups with either FLX or flaxseed oil (G9 and G10) confirming their antidepressant-like effect.

FST is commonly used behavioral despair in rat models to detect antidepressant potential by measuring the decrease in immobility periods. In the present study, female rats undergoing estradiol withdrawal show greater immobility periods in FST as compared to control animals and thus showed depression-like behavior. Administration of flaxseed oil of G9 and G10 cultivars through the postpartum period significantly enhanced escape-directed behaviors (climbing or swimming) with significant longer duration than control group (Figure 6). This active response was considered as behavioral profiles consistent with an antidepressant-like action. This depression-like response could not be explained by the change in general locomotor activity, as these rats were active in the open field test. Continual treatment with either flaxseed (G9 and G10) or FLX was able to reverse the depression-like behaviors in the FST. The improvement effect was more pronounced in G9- and FLX-treated groups.

#### 4.2.2. Effect of Oral Administration of Flaxseed Oil on the Immune Organ Index

The results of Table 3 revealed that HSP induced decrease in body weight gain and immune organ indexes. To a certain degree, the thymus index and spleen index reflect immune function in the body. Slowed body weight increase in PPD untreated females is also being used as a proxy measure of the impact of postpartum depression symptoms [46]. As compared with sham group, the thymus and spleen indexes and body weight gain in PPD rats were significantly decreased within two weeks after hormone withdrawal [28]. Administration of oil samples (G9 and G10) or FLX for two weeks significantly increased body weight gain and improved the hypofunctional immune status in thymus and spleen in PPD rats by increasing the mass of immune organs; however, flaxseed oil of G9 cultivar exhibited more pronounced effect than G10, which may be due to its higher PUFA content. These results confirmed the potential regulatory role of flaxseed oil in immune function of PPD rat. Our results are also in agreement with the previous study, which reported that omega-3 PUFA supplementation improved the performance of cognitive parameter and prevented the mood and anxiety disorders [19]. Furthermore, many animal and human studies indicated that supplementation with *ω*-3 PUFA containing products could be of great values for the development of a healthy brain, memory, and learning [28, 47].

#### 4.2.3. Effect of Oral Administration of Flaxseed Oil on the Level of Biochemical Markers in Postpartum Depression (PPD) Rat Model

Depression is multifactor disorder and its etiology includes genetics, environmental, psychological, and biological factors. Several molecular mechanisms play a role in pathogenesis of depression [48]. Although selective serotonin reuptake inhibitors as FLX and tricyclic antidepressants are indicated for the treatment of PPD, their safety is not well established on neurological development of infants during the breastfeeding period. Hence, a safe antidepressant is warranted in the treatment of PPD [49].

Corticosterone (CORT) is an important stress hormone in animals and has been involved in major depressive disorder. CORT is commonly used as an endocrinological diagnostic marker based on its elevation in chronic stress in animal models [50]. Two isoforms of monoamine oxidase (MAO-A and MAO-B) exist, with higher substrate preference of MAO-A isoform for serotonin; in addition, it is the main target for the antidepressant MAO inhibitors. MAO-A was found to regulate the metabolic degradation of serotonin and catecholamines in noradrenaline and serotonin neurons. This is thought to be the site of therapeutic action in depression [51]. In the present study, PPD rats showed significant increase in serum level of CORT and brain activity of MAO-A relative to sham rats (Table 4). This effect was nearly restored by oral administration of both oil samples (G9 and G10) as well as by FLX relative to PPD rats, which reflected the antidepressant-like potential.

The imbalance in proinflammatory cytokines plays a role in the etiopathology of mood shifts in PPD [28, 52]. Among the proinflammatory cytokines believed to be at the top of the stress-induced inflammatory cascade is the proinflammatory cytokine interleukin-1 beta (IL-1*β*), which has been shown to be involved in inflammatory responses leading to stress-related cellular damage. It was demonstrated that IL-1*β* likely plays a role in initiating the inflammatory cascade in response to stress [53]. Based on this concept, the obtained results showed that PPD induced significant increase in MAO-A activity, levels of CORT, and proinflammatory cytokines (TNF-*α*, IL-1*β*, and IL-6); however, daily administration of both flaxseed oil at a dose of 270 mg/kg b.wt and FLX to PPD groups for 2 weeks effectively decreased MAO-A, CORT and effectively suppressed the biochemical markers of inflammation, as compared to sham-operated group. These observations indicate that the PPD model of rats, which produce depression-like behavior, is related to high brain MAO-A activity, serum CORT, and proinflammatory cytokines. Therefore, flaxseed oil could be considered as a natural way to reduce the levels of MAO-A, CORT, and proinflammatory cytokines in PPD rats. This is supported by earlier study of Dhingra and Bhankher (2014) [30], who reported that antidepressant-like activity is mediated by inhibition of MAO-A activity and suppresses the production of proinflammatory cytokines. In addition, our results are in agreement with previous studies which reported elevated level of CORT in both humans [54] and rats [3] during the postpartum period. In addition, it has been confirmed that higher levels of CORT during the postpartum period showed increase in depressive-like behavior in female rats and decrease in maternal care, hippocampal cell proliferation, and body weight [55].

Clinical and experimental tests proved that the immune system is dysregulated in PPD [7, 28] and omega-3 fatty acid deficiency increases constitutive proinflammatory cytokines production in rats [56]. It was reported that proinflammatory cytokines (e.g., IL-6 and IL-8) are activated by nuclear factor *κ*B (NF*κ*B), a transcription factor. Activity of this transcription factor is affected by levels of ROS and glutamate. NF*κ*B itself increases oxidative stress in the organism and causes inflammatory reaction which can lead to neuroprogression [57]. Overproduction of proinflammatory cytokines can be related to insufficient activity of antioxidant enzymes and low level of antioxidants. Overproduction can lead to pathological changes in brain that can escalate to cognitive dysfunction or neuropsychiatric disorders [48]. However, supplementation of omega-3 fatty acids can decrease the level of CORT and proinflammatory; therefore, omega-3 fatty acids attenuate the depressive and anxiolytic effects of proinflammatory cytokines in postpartum-induced rats [3].

The present study may provide* in vivo* evidences of antidepressant-like activity mediated through inhibition of MAO-A, antioxidant, anti-inflammatory, and neuroprotective potential of flaxseed oil of Egyptian cultivars of G9 and G10 at a dose of 270 mg/kg b.wt (equivalent to a human dose of 3 g/day) for 15 days comparable to FLX as a reference antidepressant drug in PPD rat model.

#### 4.2.4. Effect of Flaxseed Oil on the Hippocampal Antioxidant Defense State

Lipid peroxidation occurs more frequently in brain tissues due its high capacity in oxygen consumption and its high content of lipids and transition metals [58]. The hippocampus is one of the few brain areas that facilitate adult neurogenesis, and disruption of that process has been implicated in many mental disorders. It is involved in learning, memory, motivational control, and emotional control. The hippocampus also mediates the hypothalamic-pituitary-adrenal axis and expresses a high number of serotonin and GABAergic receptors, making it a popular therapeutic target for antidepressant action [59]. It is particularly sensitive to insults, such as stress, ischemia, and aging [60]. Oxidative stress, an imbalance between oxidative and antioxidative systems, is implicated in depression through its free radicals by-products including reactive oxygen species (ROS) and reactive nitrogen species (RNS). Lipid peroxidation occurs when ROS production exceeds the antioxidant capacity [44]. Products of oxidative stress represent pivotal markers for detecting and measuring of depression status as well as for determining effectiveness of antidepressants [48]. In the brain tissue, antioxidant enzymes like superoxide dismutase (SOD), catalase (CAT), and the nonenzymatic endogenous reduced glutathione GSH are responsible for detoxification of ROS [61]. Lowered concentration of ascorbic acid (vit C) was observed also in patients with depression and its intravenous administration not only amplifies antidepressants efficacy, but also act as antidepressant itself [62]. Deficiency of several nonenzymatic antioxidants is connected with worsening depression severity and anxiety [62]. Changes in activity of oxidative stress-related enzymes are associated with depression and inflammation [48].

From the aforementioned discussion, it seems necessary to measure the hippocampal antioxidant defense state. The results in Table 5 demonstrated that PPD induced by HSP in rats was associated with significant decrease in hippocampal SOD and CAT activity and depletion in levels of GSH and ascorbic acid as compared with normal control rats. In addition, the markers of lipid peroxidation (MDA) and protein oxidation (PC) as well as NO were significantly elevated in PPD group, which confirmed the susceptibility of brain tissues to oxidative stress. These results suggested that decreased antioxidant enzyme activities result in accumulation of ROS and negatively correlated with severity of depression. On the contrary, oral administration of flaxseed oil cultivars (G9 or G10) at a dose of 270 mg/kg b.wt for 2 weeks to rats with PPD induced by HSP significantly restored the hippocampal redox balance state and attenuated PPD-induced oxidative damage (Table 5). The best oxidative stress prevention effect was obtained with G9, followed with FLX and then G10 in case of SOD, PC, and NO, while for enzyme activity of CAT and level of GSH and MDA, the following order was recorded: FLX > G9 > G10. Also, it should be pointed out that administration of FLX and G9 showed the same improvement degree in hippocampal content of vit C. These results are consistent with data reported on multifaceted action of omega-3 from fish oil through anti-inflammatory, antioxidative, and antiapoptotic effects which improved the learning and memory in diabetic rats [58, 63].

The potential of diet for the preservation of hippocampal function was investigated by Labrousse et al., 2012 [64], who demonstrated that the treatment with EPA/DHA for eight weeks increased the long-chain *ω*-3 PUFAs in the brain tissues, prevented cytokines expression and alteration in astrocytes morphology of the hippocampus, and enhanced the spatial memory and Fos-associated activation in the hippocampus of aged mice. Thomas et al. [60] demonstrated that dietary supplementation regime of resveratrol or docosahexaenoic acid (DHA) or their combination effectively altered the hippocampal gene expression through anti-inflammatory mechanisms. Furthermore, supplementation with EPA, DHA, and *α*-ALA prevents the cognitive impairment by maintaining membrane integrity, enhancing neurogenesis, decreasing neuroinflammation, and increasing the cerebral blood flow [65]. On the other hand, overproduction of nitric oxide (NO) is strong damaging-free radical. However, the low concentration NO is considered as an important neurotransmitter connected to pathophysiology of depression, anxiety, epilepsy, and schizophrenia. NO affects sexual and aggressive behavior and via synthesis pathway also takes part in anxious behavior, inflammation, and depression elicited by interferon alpha [66]. Ren and Chung (2007) revealed that anti-inflammatory effect of ALA is mediated through the inhibition of NO production and inducible nitric oxide synthase gene expression [67]. Elevated concentration of NO was detected in patients with depression [68]. Antidepressant-like effect can be induced by inhibition of NO synthesis in brain; inhibition of inducible nitric oxide synthase (iNOS) leads to increased effectiveness of serotonergic antidepressants and can be applied to patients suffering from drug resistant depression [69].

Furthermore, anxiety-like behaviors assessed by elevated plus maze and open field tests and depression-like behaviors evaluated by the forced swim test confirmed the antidepressant potential of tested cultivars of flaxseed oil. It should be mentioned that this study was the first study which demonstrated the efficacy of flaxseed oil of the Egyptian cultivars G9 and G10 in the treatment of postpartum depression. On the other hand, the fewer side effects of flaxseed oil compared with the classical antidepressant FLX confirm application of flaxseed oil as an alternative treatment of depression in traditional medicine.

## 5. Conclusion

In line with our hypothesis, this study supported the correlation between oxidative stress and inflammatory responses in PPD and provides* in vivo* evidences of efficacy of Egyptian cultivars of flaxseed oil G9 and G10 in alleviating depression- and anxiety-like behavior and biochemical changes in PPD-induced female rats. Accordingly, flaxseed oil may be a useful, cheap, and an alternative therapeutic agent for treating stress-related disorders such as postpartum depression. However, further studies are required to clarify this hypothesis in humans, which might support a novel preventive strategy to slow down the symptoms of PPD.

## Figures and Tables

**Table 1:** Results of GC analysis of fatty acid methyl esters of different oil samples of flaxseeds and their percentage of oil yields.

Peak	Rt^**∗**^	Comp name	Relative percentage (area%)					
			S1	S2	S3	S4	G9	G10
1	23.71	Palmitic acid methyl ester	5.98	6.47	1.97	6.16	6.44	6.16
2	24.72	Hexadecanoic acid, ethyl ester	1.1	—	5.56	—	—	—
3	28.47	Stearic methyl ester	5.19	4.95	1.76	5.64	4.65	4.95
4	29.07	Oleic acid methyl ester (9*Z*) *ω*-9	20.84	15.93	4.72	13.74	14.72	18.54
5	29.25	9-Octadecanoic acid methyl ester (9*E*)	—	1.01	3.47	—	0.97	—
6	29.90	Ethyl oleate (9*Z*) *ω*-9	1.28	0.76	9.38	1.57	0.48	—
7	30.36	10,13-Octadecadienoic acid methyl ester (10*E*, 13*E*)	11.19	12.70	3.8	13.45	12.96	11.87
8	31.17	Linoleic ethyl ester (9*Z*, 12*Z*) *ω*-6	1.11	0.96	10.52	1.63	0.74	—
9	32.04	*α*-Linolenic acid methyl ester (9*Z*, 12*Z*, 15*Z*) *ω*-3	46.70	51.31	13.47	50.85	53.88	51.74
10	32.69	Linolenic acid ethyl ester *ω*-3	6.61	5.92	45.35	6.96	5.16	6.74

Oil yield (%)			35.8	42.50	33.2	35.1	40.74	34.8

Rt^*∗*^, retention time in min. S1: Sahka 1, S2: Sakha 2, S3: Sakha 3, S4: Sakha 4, G9: Giza 9, and G10: Giza 10.

**Table 2:** The behavioral parameters recorded in elevated plus maze.

Groups	TL on 15th day (sec.)	TL on 24 hrs (sec.)	ΔTL (sec.)
Sham	41.1 ± 1.39^A^	34.9 ± 1.49^A^	6.25 ± 0.41^C^
PPD	52.1 ± 1.53^C^	49.9 ± 1.57^C^	2.25 ± 0.16^A^
PPD + G9	45.0 ± 1.17^AB^	39.4 ± 1.08^B^	5.63 ± 0.38^BC^
PPD + G10	47.3 ± 1.80^B^	42.4 ± 1.74^B^	4.88 ± 0.13^B^
PPD + FLX	44.5 ± 1.35^AB^	38.8 ± 1.10^AB^	5.75 ± 0.37^BC^

Each value represents the mean of 8 rats ± SE. In the same column, the different letters indicate a significant difference between groups using one-way ANOVA by using SPSS software (version 22) followed by Duncan's multiple-comparison test at *P* < 0.05. PPD: postpartum depression; G9 and G10: flaxseed oils from cultivars Giza 9 and Giza 10; and FLX: fluoxetine.

**Table 3:** Effect of different treatments on body weight and immune organ index.

Groups	Initial body weight (g)	Final body weight (g)	Weight gain (g)	Weight of thymus (mg)	Thymus index (mg/g)	Weight of spleen (mg)	Spleen index (mg/g)
Sham	155.4 ± 1.69^A^	211.5 ± 1.20^B^	56.1 ± 1.78^B^	195.6 ± 4.70^C^	0.93 ± 0.021^B^	769.3 ± 25.2^C^	3.64 ± 0.11^C^
PPD	157.3 ± 2.23^A^	182.6 ± 2.31^A^	25.3 ± 3.14^A^	146.8 ± 3.50^A^	0.80 ± 0.017^A^	552.6 ± 8.09^A^	3.03 ± 0.07^A^
PPD + G9	160.5 ± 1.65^A^	211.1 ± 1.09^B^	50.6 ± 1.71^B^	188.7 ± 2.60^BC^	0.89 ± 0.014^B^	733.3 ± 10.9^BC^	3.47 ± 0.05^BC^
PPD + G10	158.3 ± 2.61^A^	208.1 ± 3.16^B^	49.8 ± 3.02^B^	183.5 ± 2.20^B^	0.88 ± 0.015^B^	685.3 ± 22.5^B^	3.29 ± 0.08^B^
PPD + FLX	155.1 ± 1.25^A^	210.9 ± 2.81^B^	55.8 ± 2.48^B^	190.4 ± 3.63^BC^	0.90 ± 0.018^B^	746.9 ± 15.7^C^	3.54 ± 0.05^C^

Each value represents the mean of 8 rats ± SE. In the same column, different letters indicate a significant difference between groups using one-way ANOVA by using SPSS software (version 22) followed by Duncan's multiple-comparison test at *P* < 0.05. PPD: postpartum depression; G9 and G10: flaxseed oils from cultivars Giza 9 and Giza 10; and FLX: fluoxetine.

**Table 4:** Effect of different treatments on brain monoamine oxidase (MAO-A), serum corticosterone (CORT), and proinflammatory cytokines.

Groups	MAO-A (nmol/mg protein)	CORT (ng/ml)	TNF-*α* (pg/ml)	IL-1*β* (pg/ml)	IL-6 (pg/ml)
Sham	43.2 ± 1.62^A^	42.5 ± 0.95^A^	25.6 ± 0.99^A^	39.4 ± 0.82^A^	17.2 ± 0.90^A^
PPD	63.5 ± 1.73^D^	81.4 ± 1.90^C^	51.2 ± 1.13^C^	76.7 ± 1.22^C^	67.6 ± 2.79^D^
PPD + G9	47.3 ± 1.70^AB^	55.9 ± 1.70^B^	29.6 ± 0.76^B^	50.4 ± 1.27^B^	32.7 ± 1.98^BC^
PPD + G10	54.7 ± 2.23^C^	58.0 ± 1.88^B^	31.4 ± 0.98^B^	50.8 ± 1.35^B^	35.4 ± 1.51^C^
PPD + FLX	50.8 ± 1.87^BC^	54.7 ± 0.98^B^	29.0 ± 0.97^B^	47.7 ± 1.40^B^	27.7 ± 1.44^B^

Each value represents the mean of 8 rats ± SE. In the same column, the different letters indicate a significant difference between groups using one-way ANOVA by using SPSS software (version 22) followed by Duncan's multiple-comparison test at *P* < 0.05.

**Table 5:** Effect of different treatments on the hippocampal redox state.

Groups	Enzymatic defense system		Nonenzymatic defense system		Markers of oxidative stress		
	SOD	CAT	GSH	Vit. C	PC	MDA	NO
Sham	4.23 ± 0.09^C^	71.2 ± 1.36^C^	1.20 ± 0.02^B^	1.54 ± 0.08^B^	1.36 ± 0.05^A^	13.7 ± 0.45^A^	4.11 ± 0.16^A^
PPD	3.09 ± 0.11^A^	54.1 ± 1.74^A^	0.98 ± 0.05^A^	1.21 ± 0.02^A^	1.78 ± 0.10^B^	17.1 ± 0.47^C^	7.44 ± 0.15^C^
PPD + F5	3.90 ± 0.08^BC^	65.7 ± 1.55^B^	1.14 ± 0.03^B^	1.43 ± 0.05^B^	1.47 ± 0.06^A^	14.7 ± 0.47^AB^	4.59 ± 0.25^AB^
PPD +F6	3.81 ± 0.11^BC^	64.2 ± 1.88^B^	1.12 ± 0.03^B^	1.39 ± 0.04^B^	1.49 ± 0.06^A^	15.2 ± 0.25^B^	4.83 ± 0.16^B^
PPD + FLX	3.63 ± 0.25^B^	65.8 ± 1.75^B^	1.17 ± 0.03^B^	1.43 ± 0.02^B^	1.52 ± 0.07^A^	14.2 ± 0.29^AB^	4.70 ± 0.19^B^

Each value represents the mean of 8 rats ± SE. In the same column, the different letters indicate a significant difference between groups using one-way ANOVA by using SPSS software (version 22) followed by Duncan's multiple-comparison test at *P* < 0.05. PPD: postpartum depression; G9 and G10: flaxseed oils from cultivars Giza 9 and Giza 10; and FLX: fluoxetine.
